# Effects of A‐PRF^+^ Extract at Different Concentrations on the Metabolic Activity and Migration of Dental Pulp Stem Cells in Irreversible Pulpitis: An In Vitro Study

**DOI:** 10.1155/ijod/3582890

**Published:** 2026-07-06

**Authors:** Duyen Ngoc-Minh Tran, Minh Duc Nguyen, Ninh Hai Truong, Giang Thanh Pham, Thinh Viet Vo, Hoang Minh Lam, My Thi-Ngoc Nguyen, Ha Le-Bao Tran, Lan Thi-Quynh Ngo, Khue Ngoc Luong, Anh Thi-Nguyet Nguyen

**Affiliations:** ^1^ Odonto-Maxillo Facial Hospital, Ho Chi Minh City, Vietnam; ^2^ Faculty of Dentistry, University of Medicine and Pharmacy, Ho Chi Minh City, Vietnam, yds.edu.vn; ^3^ College of Medicine and Pharmacy, Tra Vinh University, Vinh Long City, Vietnam, tvu.edu.vn; ^4^ University of Science, Vietnam National University, Ho Chi Minh City, Vietnam, vnu.edu.vn; ^5^ Faculty of Dentistry, University of Medicine and Pharmacy, Vietnam National University, Hanoi, Vietnam, vnu.edu.vn

**Keywords:** A-PRF^+^, dental pulp stem cells, irreversible pulpitis, metabolic activity, migration, platelet concentrates, regeneration, viability

## Abstract

**Background:**

Vital pulp therapy (VPT) in mature permanent teeth with irreversible pulpitis represents a paradigm shift toward preserving pulp vitality. Advanced platelet‐rich fibrin plus (A‐PRF^+^) is a bioactive scaffold capable of releasing high concentrations of growth factors; however, its concentration‐dependent effects on inflamed pulp‐derived stem cells remain unclear. This study aimed to evaluate the concentration‐dependent effects of A‐PRF^+^ and identify a biologically favorable concentration range for supporting the functional activity of dental pulp stem cells isolated from teeth with irreversible pulpitis.

**Methods:**

IP‐DPSCs were isolated from inflamed pulp tissues and characterized by flow cytometry and trilineage differentiation assays. A‐PRF^+^ was prepared using the low‐speed centrifugation concept (LSCC). Cells were exposed to sequential dilutions of A‐PRF^+^ extracts (100%, 50%, and 25%). Cell metabolic activity was assessed using the MTT assay. Cell migration was evaluated using a scratch assay, and wound closure rates were quantified with ImageJ software. Statistical analysis was performed using one‐way or two‐way ANOVA with appropriate post‐hoc tests.

**Results:**

Flow‐cytometric analysis showed high expression of MSC‐associated markers (CD44, CD73, and CD90) and negligible expression of hematopoietic markers, while differentiation along osteogenic, chondrogenic, and adipogenic lineages was observed following induction culture. A‐PRF^+^ exhibited a biphasic concentration‐dependent effect. The 25% extract significantly enhanced cell migration, achieving a wound closure rate of 27.09 ± 4.03%, compared with 13.16 ± 4.50% in the 100% extract group (*p* = 0.013). Undiluted A‐PRF^+^ showed inhibitory effects on both migration and metabolic activity.

**Conclusion:**

A‐PRF^+^ exerted concentration‐dependent effects on IP‐DPSCs, with diluted preparations supporting greater migration and metabolic activity than undiluted extracts. These findings suggest the existence of a biologically favorable concentration range for A‐PRF^+^ and provide a rationale for further investigation of concentration optimization in regenerative endodontic applications.

## 1. Introduction

Recent advances in regenerative endodontics have shifted the clinical paradigm from conventional pulpectomy toward vital pulp therapy (VPT), even in teeth diagnosed with irreversible pulpitis. Mature permanent teeth with irreversible pulpitis have traditionally been treated by complete pulpectomy, a procedure that eliminates pulp vitality and may compromise the long‐term integrity of the tooth. Contemporary regenerative approaches seek to preserve pulp vitality by harnessing the intrinsic healing potential of the dentin–pulp complex through the use of bioactive materials and scaffolds [1, 2].

Among these, advanced platelet‐rich fibrin plus (A‐PRF^+^) has attracted increasing attention. Prepared using the low‐speed centrifugation concept (LSCC), A‐PRF^+^ provides a fibrin scaffold enriched with platelets, leukocytes, and bioactive molecules, together with a sustained release of growth factors. Promising outcomes have been reported in bone regeneration and regenerative endodontic procedures involving immature teeth [3, 4]. However, its biological effects in the context of VPT for mature teeth remain incompletely understood.

The response of pulp‐derived stem cells is likely to play an important role in the success of such therapies. Previous studies have demonstrated that stem cells isolated from inflamed pulp tissues retain mesenchymal stem cell characteristics and self‐renewal potential despite exposure to a chronic inflammatory microenvironment [5]. Although PRF‐derived products have been reported to stimulate cellular proliferation and migration, the biological response to highly concentrated preparations remains unclear. Excessive concentrations of growth factors may induce receptor saturation, negative feedback signaling, or cellular stress, thereby attenuating the desired cellular response. Such biphasic dose–response patterns have been described in various biological systems and may contribute to the concentration‐dependent effects observed with platelet‐derived products.

Therefore, identifying a biologically favorable concentration range is important for the potential application of A‐PRF^+^ in inflamed pulps. The present study aimed to evaluate the concentration‐dependent effects of A‐PRF^+^ extracts on the viability, migration, and metabolic activity of inflamed pulp‐derived cells isolated from teeth with irreversible pulpitis. Using a sequential dilution model designed to explore the potential influence of changing growth‐factor availability over time, we sought to identify a concentration range capable of supporting the functional activity of inflamed pulp‐derived cells.

## 2. Methods

The study protocol was approved by the Ethical Committee of the Faculty of Dentistry, University of Medicine and Pharmacy at Ho Chi Minh City, Vietnam (Number 2254/ĐHYD‐HĐĐĐ).

### 2.1. Cell Isolation and Characterization

IP‐DPSCs were isolated from two donors requiring endodontic treatment for irreversible pulpitis. Briefly, pulp tissues were harvested from the coronal pulp chamber under aseptic conditions and placed in phosphate‐buffered saline (PBS) containing 4% (v/v) penicillin–streptomycin solution. Cells were isolated using the outgrowth method according to a previously described protocol for inflamed pulp‐derived stem cells [5]. Cells were cultured in a complete medium (DMEM/F12 supplemented with 10% fetal bovine serum and 1% antibiotics) at 37°C in a humidified atmosphere containing 5% CO_2_. Cells from passage 4 were used for all experiments.

Cells were characterized by flow cytometry and multilineage differentiation assays. Surface marker expression was analyzed using antibodies against CD44, CD73, CD90, CD105, CD14, CD34, CD45, and HLA‐DR. Multipotency was evaluated through osteogenic, chondrogenic, and adipogenic differentiation under lineage‐specific induction conditions.

### 2.2. Flow Cytometric Analysis

Passage 3 cells were harvested and incubated with fluorochrome‐conjugated antibodies according to the manufacturers’ instructions. After washing, samples were analyzed using a BD FACSMelody Cell Sorter flow cytometer, and data were processed using FlowJo software. Marker expression was reported as the percentage of positive cells after the exclusion of debris and dead cells.

### 2.3. Multilineage Differentiation Assays

To assess multipotency, passage 3 cells were cultured in osteogenic, chondrogenic, and adipogenic induction media according to the manufacturer’s protocols [6]. Multipotency was assessed through osteogenic, chondrogenic, and adipogenic induction using commercially available differentiation kits. Osteogenic and chondrogenic differentiation were evaluated after 28 days, whereas adipogenic differentiation was evaluated after 14 days.

### 2.4. A‐PRF^+^ Extract Preparation

A‐PRF^+^ was prepared by using the LSCC. Venous blood was collected and centrifuged at 1300 rpm for 8 min (DUO Quattro, France). The resulting A‐PRF^+^ gel was separated and compressed to remove the exudate. To obtain extracts, A‐PRF^+^ membranes were incubated in a basal medium (DMEM‐F12) at a ratio of 0.2 g/mL (as per ISO 10993‐12) for 24 h at 37°C. The supernatant was harvested as the 100% extract, which was then sequentially diluted with basal medium to obtain 50% and 25% concentrations.

### 2.5. Cell Viability and Metabolic Activity Assays

Cell viability and metabolic activity, frequently used as a surrogate indicator of proliferation in PRF‐related studies, were assessed using the MTT assay. For viability, IP‐DPSCs were seeded at 10^4^ cells/well in 96‐well plates. After 24 h of attachment, cells were exposed to A‐PRF^+^ extracts for 24 h. For metabolic activity, cells were seeded at 2 × 10^3^ cells/well and incubated in A‐PRF^+^ conditions for 1, 3, 5, 7, and 9 days. Complete medium (CM) and basal medium containing 20% DMSO served as controls. At each time point, MTT solution (0.5 mg/mL) was added and incubated for 4 h. Formazan crystals were dissolved in DMSO, and absorbance was measured at 570 nm.

### 2.6. Scratch Wound Healing Assay

Cell migration was quantified using a scratch assay. IP‐DPSCs were seeded into 6‐well plates (2.5 × 10^5^ cells/well) until confluence, followed by 24 h of starvation in basal medium. A vertical scratch was introduced using a 1‐mL pipette tip. After washing away debris, cells were treated with A‐PRF^+^ extracts or control media (CM and basal medium). Digital images were captured at 0 and 24 h using an inverted microscope. The cell‐free area was measured using ImageJ software (Version 1.53, NIH, USA). The Polygon tool was used to manually outline wound margins across three different fields per well. The wound closure (%) was calculated using the following formula:

Wound closure %=Area0h−Area24h/Area0h×100

All measurements were performed by a single blinded investigator to minimize bias.

### 2.7. Sequential Dilution Model

To approximate progressive changes in growth‐factor availability, cells were initially exposed to 100% extract. The concentration was subsequently reduced to 50% and then 25% at predetermined time points during the culture period according to the predefined experimental schedule.

### 2.8. Statistical Analysis

All experiments were performed in triplicate for each donor. Because only two biological donors were available, the statistical analyses should be interpreted as exploratory and primarily reflect within‐study comparisons rather than population‐level inferences. To ensure objective quantification of the migration assay, ImageJ software (Version 1.53, NIH, USA) was used to measure the wound areas. After confirming normality via the Shapiro–Wilk test, differences between groups were evaluated using one‐way ANOVA, followed by Tukey’s post‐hoc test. For time‐course data, two‐way ANOVA (group × time) with Dunnett’s post‐hoc multiple‐comparison test was performed to compare each treatment group with the negative control at each time point. A *p*‐value < 0.05 was considered statistically significant.

## 3. Results

### 3.1. Characterization of Isolated Cells

**Figure 1:**
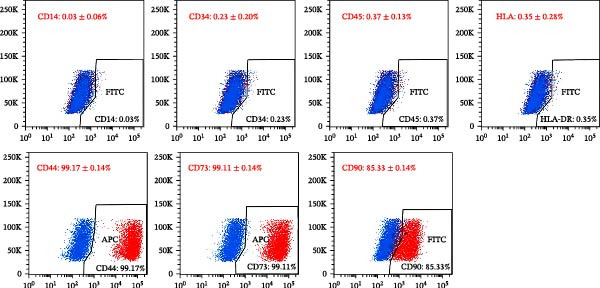
Flow‐cytometric characterization of isolated cell population.

Flow‐cytometric analysis demonstrated high expression of CD44 (99.17 ± 0.14%), CD73 (99.11 ± 0.14%), and CD90 (85.33 ± 0.14%), with minimal expression of CD14 (0.03 ± 0.06%), CD34 (0.23 ± 0.20%), CD45 (0.37 ± 0.13%), and HLA‐DR (0.35 ± 0.28%) (Figure 1 and Table 1). CD105 expression was not detected in the analyzed cell population. In addition, cells exhibited osteogenic, chondrogenic, and adipogenic differentiation following lineage‐specific induction cultures.

**Table 1:** Immunophenotypic profile of the isolated cell population.

Marker	Positive cells (%)
CD44	99.17 ± 0.14
CD73	99.11 ± 0.14
CD90	85.33 ± 0.14
CD105	Negative/not detected
CD14	0.03 ± 0.06
CD34	0.23 ± 0.20
CD45	0.37 ± 0.13
HLA‐DR	0.35 ± 0.28

### 3.2. Cell Viability Under A‐PRF^+^ Extracts

**Figure 2:**
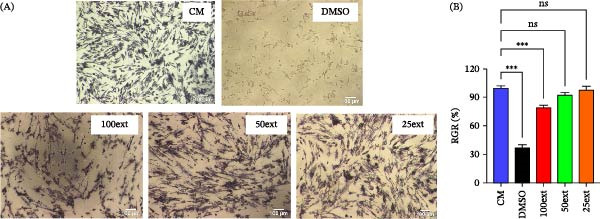
MTT assay for cell viability under different percentages of A‐PRF^+^ extracts. (A) Microscopic observation of crystal violet formation after MTT incubation. (B) Comparison of the relative growth rates of the experimental groups compared with the positive control group. CM: complete medium as positive control. DMSO: 20% DMSO treatment as negative control. 100ext, 50ext, and 25ext: 100%, 50%, and 25% of A‐PRF^+^ extracts, respectively. Data were analyzed using ANOVA followed by Tukey’s post‐hoc test; statistically significant differences are indicated as  ^∗∗∗^ *p* < 0.001; ns: not significant.

The MTT assay revealed a concentration‐dependent effect of A‐PRF^+^ on IP‐DPSCs viability. In the positive control (CM), robust formation of purple formazan crystals indicated high cell viability, while the negative control (20% DMSO) showed significant cytotoxicity (Figure 2A). Regarding the experimental groups, cell viability increased progressively as the A‐PRF^+^ extract was diluted. The 100% extract resulted in sparse viable cells, achieving only 78.21% relative growth compared to that of CM. Conversely, lowering the concentration significantly improved cell survival. The 25% extract exhibited the highest viability (98.15%), which was significantly superior to the 100% group (*p* < 0.05) and comparable to the CM control (*p* > 0.05) (Figure 2B).

Representative histograms show the expression of mesenchymal stromal cell‐associated markers (CD44, CD73, and CD90) and minimal expression of hematopoietic markers (CD14, CD34, CD45, and HLA‐DR).

### 3.3. Quantitative Migration Analysis (Scratch Assay)

**Figure 3:**
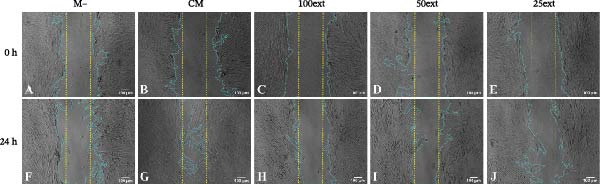
Scratch assay for cell migration in different percentages of A‐PRF^+^ extracts. M−: Basal medium. CM: Complete medium. 100ext, 50ext, 25ext: 100%, 50%, and 25% of A‐PRF^+^ extracts, respectively. (A, F) Basal medium as negative control for minimal cell migration at 0 and 24 h. (B, G) Complete medium as positive control for increasing cell migration at 0 and 24 h. (C, H) 100% A‐PRF^+^ extract at 0 and 24 h. (D, I) 50% A‐PRF^+^ extract at 0 and 24 h. (E, J) 25% A‐PRF^+^ extract at 0 and 24 h.

The scratch wound healing assay demonstrated a concentration‐dependent effect of A‐PRF^+^ extracts on IP‐DPSC migration. Representative images obtained at 0 h (Figure 3A–E) and 24 h (Figure 3F–J) illustrate the distinct migration patterns observed among the experimental groups. At 24 h, the 25% extract demonstrated the greatest migratory response, achieving a wound closure rate of 27.09 ± 4.03%, which was significantly higher than the 100% extract group (13.16 ± 4.50%, *p* = 0.013) (Table 2, Figure 3). The wound closure rate in the 100% extract group (13.16 ± 4.50%) was only slightly higher than that observed in the negative control group (6.59 ± 5.10%, ns). The negative control group (M−) showed minimal wound closure over the 24‐h observation period (*p* > 0.05). In this group, morphological changes and reduced cell density were observed at the scratch periphery (Figure 3F). In contrast, 50% and 25% extracts promoted significantly higher cell distribution within the wound area (Figure 3I,J).

**Table 2:** Quantitative migration analysis of IP‐DPSCs (scratch assay).

Experimental group	Wound closure rate (%) (Mean ± SD)	*p*‐Value (vs. 100% extract)	*p*‐Value (vs. 50% extract)	*p*‐Value (vs. 25% extract)
Negative control (M−)	6.59 ± 5.10	ns	*p* = 0.036	*p* < 0.001
Positive control (CM)	74.78 ± 2.70	*p* < 0.001	*p* < 0.001	*p* < 0.001
100% A‐PRF^+^ extract	13.16 ± 4.50	—	ns	*p* = 0.013
50% A‐PRF^+^ extract	18.27 ± 3.63	ns	—	ns
25% A‐PRF^+^ extract	27.09 ± 4.03	*p* = 0.013	ns	—

### 3.4. Metabolic Activity Under Sequential Dilution Model

**Figure 4:**
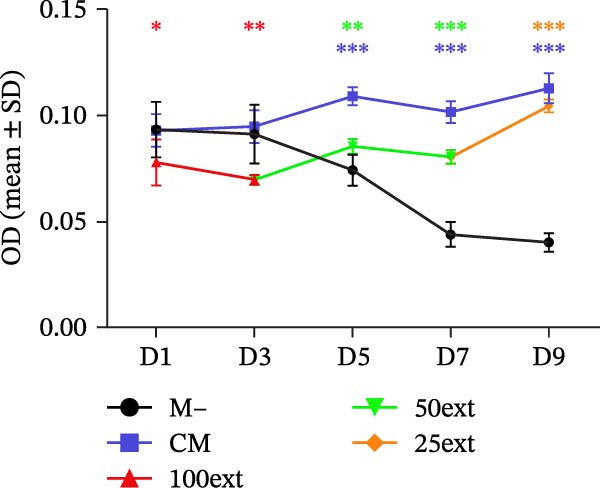
Experimental design of the sequential dilution model used in the metabolic activity assay. M−: Basal medium. CM: Complete medium. 100ext, 50ext, 25ext: 100%, 50%, and 25% of A‐PRF^+^ extracts, respectively. Two‐way ANOVA (group × time) with Dunnett’s post‐hoc test; statistically significant differences compared with the negative control group ( ^∗^ *p* < 0.05;  ^∗∗^ *p* < 0.01;  ^∗∗∗^ *p* < 0.001).

To simulate the temporal changes in growth‐factor availability, a sequential dilution model was employed. As shown in Figure 4, exposure to the 100% extract was associated with reduced metabolic activity between Days 1 and 3. Following dilution to 50%, OD values gradually increased, and a further increase was observed after dilution to 25% on Day 9. While cells in the basal medium (M−) suffered significant death after Day 3, those in the sequential A‐PRF^+^ dilution model maintained a growth pattern similar to that of the positive control.

**Figure 5:**
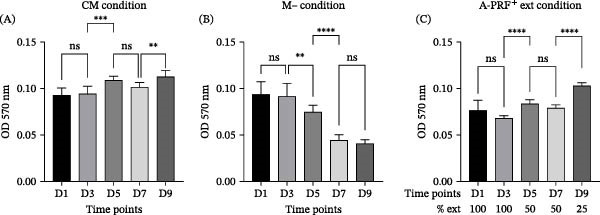
MTT‐based metabolic response of IP‐DPSCs under the sequential dilution model. (A) CM condition. (B) M− condition. (C) A‐PRF^+^ extract condition. CM: complete medium. M−: basal medium. ext: extracts of A‐PRF^+^.  ^∗^,  ^∗∗^,  ^∗∗∗^, and  ^∗∗∗∗^: significant differences. ns (not significant): *p*‐value > 0.05.

The MTT assay was also used to evaluate the metabolic response of IP‐DPSCs under the sequential dilution model (Figure 5). Cell growth was recorded in a CM (Figure 5A, CM condition), indicated by a gradually increased OD value during the culture time points. In the basal medium (Figure 5B, M− condition), although OD values were comparable to those of the CM condition during the first 3 days, cell death with a significant decrease in OD values was shown on Days 5, 7, and 9.

In A‐PRF^+^ extract conditions (Figure 5C), a representative model of diluting the bioactive factor from A‐PRF^+^ after implantation into the pulp chamber was proposed for examining IP‐DPSCs proliferation.

The cells were cultured in a 100% A‐PRF^+^ extract for 3 days, followed by a 50% A‐PRF^+^ extract until Day 7. During the last days of culture, IP‐DPSCs were treated with a 25% A‐PRF^+^ extract and terminally evaluated at Day 9. Similar to the viability and migration findings, exposure to the 100% A‐PRF^+^ extract was associated with reduced metabolic activity during the early culture period. Following dilution to 50%, metabolic activity increased and continued to rise after further dilution to 25% on Day 9. These findings indicate that lower A‐PRF^+^ concentrations were associated with improved cellular metabolic responses under the experimental conditions used in this study.

## 4. Discussion

The clinical success of VPT in teeth with irreversible pulpitis depends largely on the ability of the resident pulp‐derived stem cells to survive and function within an inflammatory microenvironment. In the present study, A‐PRF^+^ extracts exerted a clear concentration‐dependent effect on IP‐DPSCs. Undiluted extracts were associated with reduced viability, migration, and metabolic activity, whereas lower concentrations, particularly 25%, were more favorable for cellular function.

Flow cytometric and differentiation analyses demonstrated that the isolated cells exhibited characteristics consistent with mesenchymal stem cells. The cells exhibited high expression of MSC‐associated markers (CD44, CD73, and CD90) and minimal expression of hematopoietic markers (CD14, CD34, CD45, and HLA‐DR), and osteogenic, chondrogenic, and adipogenic differentiation were observed following lineage‐specific induction. These findings support the mesenchymal stem cell phenotype of the inflamed pulp‐derived cell population used in this study. Although differentiation was observed under lineage‐specific induction conditions, quantitative assessment of lineage‐specific markers was not performed and should be considered in future studies. CD105 expression was not detected in the present cell population. Although CD105 is commonly included among the markers used to characterize mesenchymal stem cells, reduced or absent CD105 expression has been reported in dental pulp‐derived cell populations obtained from inflamed tissues. Chronic inflammation may alter the immunophenotypic profile of pulp‐derived stromal cells without necessarily abolishing their functional properties. In the present study, the observed multilineage differentiation capacity and expression of CD44, CD73, and CD90 suggest that the isolated cells retained stem/stromal cell characteristics despite the absence of detectable CD105 expression.

The biphasic response observed in the present study is consistent with the concept of hormesis, whereby biological responses vary according to dose, and excessively high concentrations may become less favorable than lower concentrations [7, 8]. A‐PRF^+^ prepared using the LSCC technique has been shown to release higher levels of growth factors such as TGF‐β1, PDGF‐BB, and VEGF than conventional PRF preparations [9, 10]. Although these mediators are essential for tissue repair, excessively high local concentrations may generate unfavorable signaling conditions that ultimately limit cellular activity.

In addition to growth‐factor signaling, the high protein content of undiluted extracts may influence the physicochemical characteristics of the culture environment, including osmotic balance and nutrient availability, thereby contributing to cellular stress [11]. In contrast, the 25% extract supported greater cell viability and migration, suggesting that lower concentrations may be more favorable for maintaining cellular activity. Similar concentration‐dependent effects have been reported in stem cells from the apical papilla, where diluted A‐PRF^+^ preparations promoted more favorable cellular behavior than undiluted extracts [12]. The data showed that both donor cell lines followed a similar pattern. Although the sample size was limited to two donors, both donor‐derived cell populations demonstrated the same overall concentration‐dependent pattern, with reduced activity at a 100% extract and improved migration and viability at lower concentrations.

Because growth‐factor release from PRF is expected to change over time, a sequential dilution model was used in the proliferation experiment. Growth factor availability following clinical application of PRF is not static but gradually changes over time as the fibrin matrix undergoes degradation and its bioactive components are released [10]. Within this model, metabolic activity initially declined during exposure to the undiluted extract but progressively recovered as the extract concentration was reduced. These findings suggest that lower concentrations of A‐PRF^+^ may be more compatible with sustained cellular activity than continuous exposure to highly concentrated preparations. However, it should be noted that the MTT assay reflects cellular metabolic activity and cannot directly distinguish cell proliferation from other processes affecting the mitochondrial function. Therefore, the observed changes in OD values should be interpreted as alterations in cellular metabolic responses rather than definitive evidence of proliferation.

The present study has several limitations, including the small number of biological donors, the absence of a healthy DPSC comparison group, the lack of growth‐factor quantification, and the absence of mechanistic analyses. In addition, because mitomycin C pretreatment was not used, wound closure may have reflected contributions from both migration and proliferation. Odontogenic differentiation markers and mineralization assays under A‐PRF^+^ treatment conditions were also not evaluated. Future studies addressing these limitations are needed to determine whether the observed concentration‐dependent effects translate into enhanced regenerative outcomes.

## 5. Conclusion

This study demonstrated a concentration‐dependent biphasic response of pulp‐derived stem cells isolated from teeth with irreversible pulpitis to A‐PRF^+^ extracts. While undiluted (100%) extracts were associated with reduced cellular metabolic activity and migration, dilution to 25% significantly enhanced these functional responses.

These findings suggest that lower concentrations of A‐PRF^+^ may provide a more favorable biological environment for supporting the functional activity of inflamed pulp‐derived stem cells. Within the limitations of this in vitro study, the results contribute to the growing evidence that optimization of growth‐factor concentration may be an important consideration in the future development of VPT and regenerative endodontic strategies.

## Author Contributions

**Duyen Ngoc-Minh Tran**: conceptualization, methodology, investigation, data curation, writing – original draft. **Minh Duc Nguyen**: investigation, data curation, validation. **Ninh Hai Truong**: investigation, formal analysis. **Giang Thanh Pham**: investigation, visualization. **Thinh Viet Vo**: methodology, validation. **Hoang Minh Lam**: investigation, resources. **My Thi-Ngoc Nguyen**: investigation, data curation. **Ha Le-Bao Tran**: investigation. **Lan Thi-Quynh Ngo**: supervision, validation. **Khue Ngoc Luong**: formal analysis, writing – review and editing. **Anh Thi-Nguyet Nguyen**: conceptualization, supervision, funding acquisition, writing – review and editing.

## Funding

This research was funded by the University of Medicine and Pharmacy at Ho Chi Minh City (Contract Number 246/2025/HĐ‐ĐHYD, dated April 28, 2025).

## Disclosure

No identifiable personal data or images are presented in this manuscript. All authors have read and approved the final version of the manuscript. The corresponding author had full access to all study data and takes responsibility for the integrity of the data and the accuracy of the data analysis. The funding body had no role in the design of the study, data collection, analysis, interpretation of data, or manuscript preparation.

## Ethics Statement

The study protocol involving the collection of human blood and dental pulp tissue was reviewed and approved by the Ethical Committee of the Faculty of Dentistry, University of Medicine and Pharmacy at Ho Chi Minh City, Vietnam (Reference Number 2254/ĐHYD‐HĐĐĐ).

## Consent

Written informed consent was obtained from all participants prior to sample collection. All procedures were performed in accordance with the ethical standards of the institutional and/or national research committee and with the Declaration of Helsinki and its later amendments.

## Conflicts of Interest

The authors declare no conflicts of interest.

## Data Availability

The datasets generated and/or analyzed during the current study are available from the corresponding author upon reasonable request.
